# Oncological Treatment Considerations Differ across Surgical Subspecialties Treating Malignant Peripheral Nerve Sheath Tumors: An International Survey

**DOI:** 10.1155/2020/6406439

**Published:** 2020-02-27

**Authors:** Enrico Martin, Willem-Bart M. Slooff, Winan J. van Houdt, Thijs van Dalen, Cornelis Verhoef, J. Henk Coert

**Affiliations:** ^1^Department of Plastic and Reconstructive Surgery, University Medical Center Utrecht, Utrecht, Netherlands; ^2^Department of Neurosurgery, University Medical Center Utrecht, Utrecht, Netherlands; ^3^Department of Surgical Oncology, Netherlands Cancer Institute, Amsterdam, Netherlands; ^4^Department of Surgical Oncology, University Medical Center Utrecht, Utrecht, Netherlands; ^5^Department of Surgery, Diakonessenhuis Utrecht, Utrecht, Netherlands; ^6^Department of Surgical Oncology, Erasmus Medical Center Cancer Institute, Rotterdam, Netherlands

## Abstract

**Background:**

Malignant peripheral nerve sheath tumors (MPNSTs) are rare and aggressive soft tissue sarcomas (STS) that, because of their origin, are operated by several surgical subspecialties. This may cause differences in oncologic treatment recommendations based on presentation. This study investigated these differences both within and between subspecialties.

**Methods:**

A survey was distributed among several (inter)national surgical societies. Differences within and between subspecialties were analyzed by *χ*^2^-tests.

**Results:**

In total, 30 surgical oncologists, 30 neurosurgeons, 85 plastic surgeons, and 29 “others” filled out the survey. Annual caseload, tumor sites operated, and fellowship training differed significantly between subspecialties. While most surgeons agreed upon preoperative use of MRI, the use of radiological staging and FDG-PET use differed between subspecialties. Surgical oncologists agreed upon core needle biopsies as an ideal type of biopsy while other subspecialties differed in opinion. On average, 53% of surgeons always consider preservation of function preoperatively, but 42% would never perform less extensive resections for function preservation. Respondents agreed that radiotherapy should be considered in tumor sizes >10 cm, microscopic, and macroscopic positive margins. A preferred sequence of radiotherapy administration differed between subspecialties. There was no consensus on indications and sequence of administration of chemotherapy in localized disease.

**Conclusion:**

Surgical oncologists generally agree on preoperative diagnostics; other subspecialties do not. Considering the preservation of function differed among all subspecialties. Surgeons do agree on some indications for radiotherapy, yet the use of chemotherapy in localized MPNSTs lacks consensus. A preferred sequence of multimodal therapy differs between and within surgical subspecialties, but surgical oncologists prefer neoadjuvant radiotherapy.

## 1. Introduction

Malignant peripheral nerve sheath tumors (MPNSTs) are aggressive soft tissue sarcomas (STS) that can occur at any anatomical site [1]. Approximately 25–50% of all patients are known to have neurofibromatosis type 1 (NF1) [2–6]. The diagnosis of an MPNST can be difficult as patients may present with similar symptoms compared to their benign counterparts, and MRI studies cannot distinguish a malignancy with high precision [7–9]. This can especially be troublesome in patients with NF1 that develop multiple benign nerve sheath tumors.

Surgical resection is the only curative treatment option in localized MPNSTs [4, 10]. Radiotherapy has an important role in decreasing local recurrence rates but does not affect survival [4, 11, 12]. The exact role for chemotherapy is also subject to controversy but is advocated by some as adjuvant treatment in large and deep MPNSTs [13, 14]. Unfortunately, despite curative aims of aggressive treatment including clear surgical margins, MPNSTs regularly recur and metastasize in up to 60% of patients [2–4, 15, 16].

MPNSTs are rare tumors and exact treatment strategies may differ between surgeons because patients can present at different surgical subspecialties due to their origin in nervous tissue and occurrence in NF1. While surgical oncologists consider MPNSTs as part of their sarcoma population requiring radical excision [17, 18], plastic surgeons and neurosurgeons operating peripheral nerve lesions regard them as a malignant form of nerve sheath tumor, which is treated by nerve-sparing surgery [19, 20]. Such a difference in perspective could affect clinical decision-making. This study investigated treatment recommendations and differences in opinions between surgical subspecialties treating MPNSTs on preoperative diagnostics, surgical decision-making, and the use of multimodal therapy in localized MPNSTs.

## 2. Methods

### 2.1. Study Design and Survey Instrument

A survey was constructed by two authors (EM and JHC) and tested internally with all coauthors from different surgical subspecialties. A secure electronic data capturing tool (REDCap) provided by the Dutch Plastic Surgery Society (NVPC) was used to construct the survey. This study is part of a larger survey addressing both oncological and reconstructive treatment considerations for localized MPNST. A total of 18 questions (30 in total) were used for this study, of which seven were for demographical purposes. The complete survey can be found in the Supplementary Materials. Approval for this study was obtained from our institutional review board.

### 2.2. Study Population

Several national and international surgical societies were asked to distribute the survey among their members with an accompanying text explaining the purpose of the research. Surgeons involved in the surgical management of MPNSTs were asked to fill out the survey. A reminder e-mail was sent thereafter. The survey was sent to the members of the Dutch Society of Surgical Oncology (NVCO), the Dutch Society for Surgery of the Hand (NVVH), the peripheral nerve section of the Dutch Society for Neurosurgery (NVVN), the American Society for Peripheral Nerve (ASPN), the peripheral nerve section of the European Association of Neurosurgical Societies (EANS), and the Soft Tissue and Bone Sarcoma Group of the European Organization for Research and Treatment of Cancer (EORTC). Survey responses were filled out anonymously and no personal identifying data was inquired.

### 2.3. Statistical Analysis

Responses were summarized per surgical subspecialty: oncologic surgery, neurosurgery, plastic surgery, and other surgical subspecialties. Differences were calculated with χ^2^-tests for categorical data. *p* values <0.05 were considered statistically significant. Statistical analyses and data visualization were conducted using R version 3.6.0 (R Core Team, 2019).

## 3. Results

### 3.1. Demographics of Survey Responders

**Figure 1:**
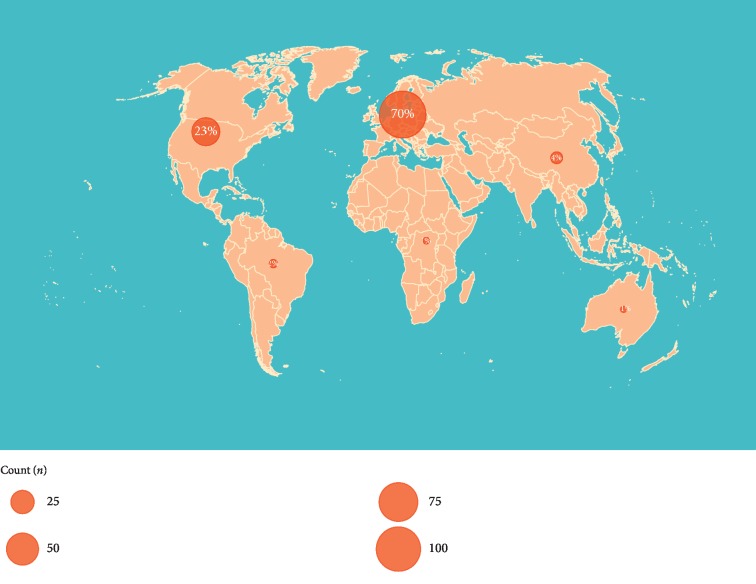
World map showing the geographical distribution of survey respondents per continent. The surface of the bubbles corresponds to the number of respondents.

In total, 174 respondents filled out the survey: 30 surgical oncologists, 30 neurosurgeons, 85 plastic surgeons, and 29 surgeons from other surgical subspecialties. Most respondents were European (Figure 1). The “other” surgical subspecialty group consisted mainly of nononcologic orthopedic surgeons and general surgeons with a hand surgery subspecialization. The largest proportion of surgeons had less than 10 years of experience as a consultant surgeon (38%, Table 1). Fellowship experience differed between subspecialties (*p* < 0.001); surgical oncologists commonly had completed a sarcoma fellowship (85%), while other respondents more commonly did a fellowship in peripheral nerve surgery (32–56%). The highest caseloads were performed by surgical oncologists (*p* < 0.001). The majority of respondents operated extremity site tumors (87%, *p* > 0.05), but most other tumor sites differed between surgical subspecialties.

### 3.2. Preoperative Diagnostics

**Figure 2:**
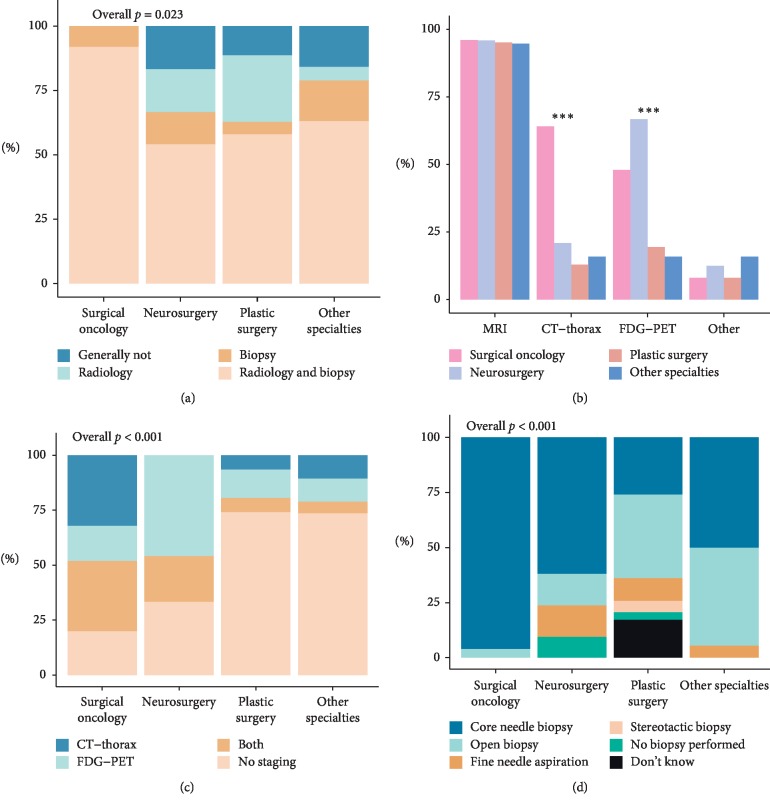
Preoperative diagnostics performed. (a) Overall preoperative diagnostics per surgical subspecialty. (b) Percentage per surgical subspecialty of different imaging techniques used. (c) Use of preoperative staging modalities per surgical subspecialty. (d) Preferred type of biopsy per surgical subspecialty. *p* values: ^*∗∗∗*^ ≤ 0.001.

Opinions regarding preoperative work-up of soft tissue tumors that may originate from peripheral nerves differ between surgical subspecialties (Figure 2). The majority of respondents would perform radiological imaging and a biopsy before operating (65%), and surgical oncologists strongly agreed on this (92%, *p* < 0.05). Regarding preoperative imaging studies, surgeons agreed that an MRI is necessary (95%, *p* > 0.05). FDG-PET scans which can be used both for staging and possible differentiation of benign and malignant lesions are more commonly performed by neurosurgeons (67%) and surgical oncologists (48%, *p* < 0.05). The preoperative staging was carried out by 44% of respondents, most commonly by surgical oncologists (80%, *p* < 0.001). A CT-thorax is used by 25%, of which more than half would be in conjunction with an FDG-PET scan. A total of 10% would also carry out other radiologic diagnostics preoperatively. Preferred type of biopsy differed significantly between the surgical subspecialties (*p* < 0.001). Overall, a core needle biopsy was the preferred type of biopsy, especially among surgical oncologists (96%). Plastic surgeons and “other” surgeons commonly also preferred open biopsies. Plastic surgeons were also most likely not to have a preferred biopsy technique (17%). Respondents that did not regard a preoperative biopsy necessary commonly reported that they considered the chances of tumor spread too high and would therefore directly proceed to surgery.

### 3.3. Surgical Treatment and Postoperative Morbidity

**Figure 3:**
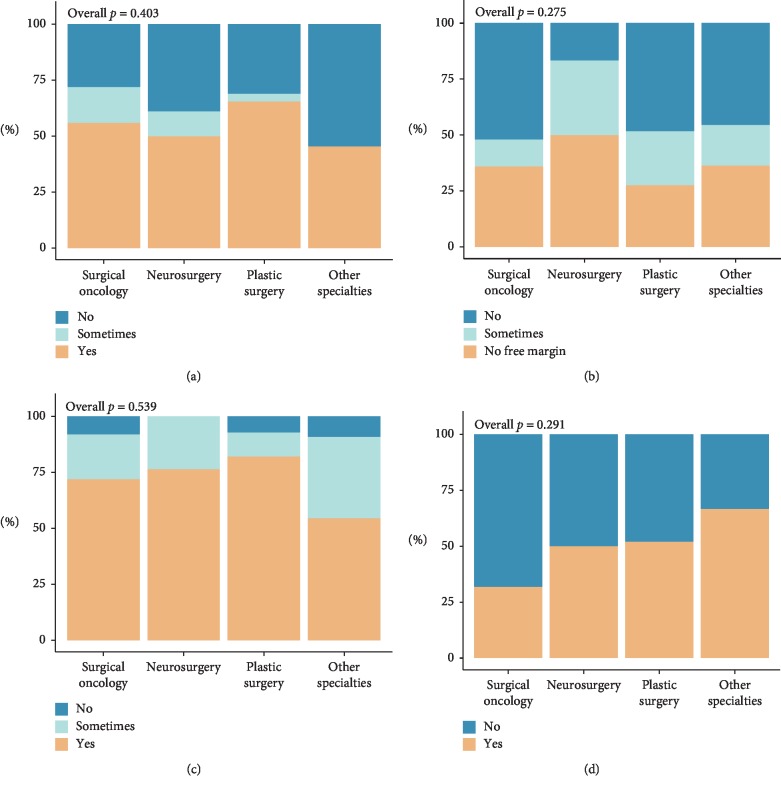
Surgical considerations per surgical subspecialty. (a) Considering the preservation of function preoperatively. (b) Performing less extensive resections to preserve function. (c) Look for originating nerve intraoperatively. (d) Resecting more nerve may lead to a decrease in recurrences.

On average, 53% of all respondents always consider preservation of function before performing a resection; most commonly plastic surgeons did so (66%, *p* > 0.05, Figure 3). Less than 8% would consider preservation of function given particular circumstances: based on localization (*n*=3), in low-grade MPNSTs (*n*=1), in case it does not interfere with oncological resection (*n*=1), when multiple lesions are present (*n*=1), or if a main nerve bundle is separable from the tumor (*n*=1). Contrarily, 42% of all surgeons would never perform less extensive resections to preserve functionality and possibly compromise oncological results, and this did not differ between surgical subspecialties (*p* > 0.05). Others would only resect less if achieving free margins was not presumed feasible (36%), while a minority would consider it in other cases as well (22%). The majority of respondents always look for the nerve of origin preoperatively (74%). In the hypothetical situation of a microscopically complete resectable MPNST, 47% of respondents had the opinion that there is a beneficial effect of resecting more of the originating nerve to decrease local recurrence as microscopic satellite lesions within or along the nerve may be present.

### 3.4. Radiotherapy

**Figure 4:**
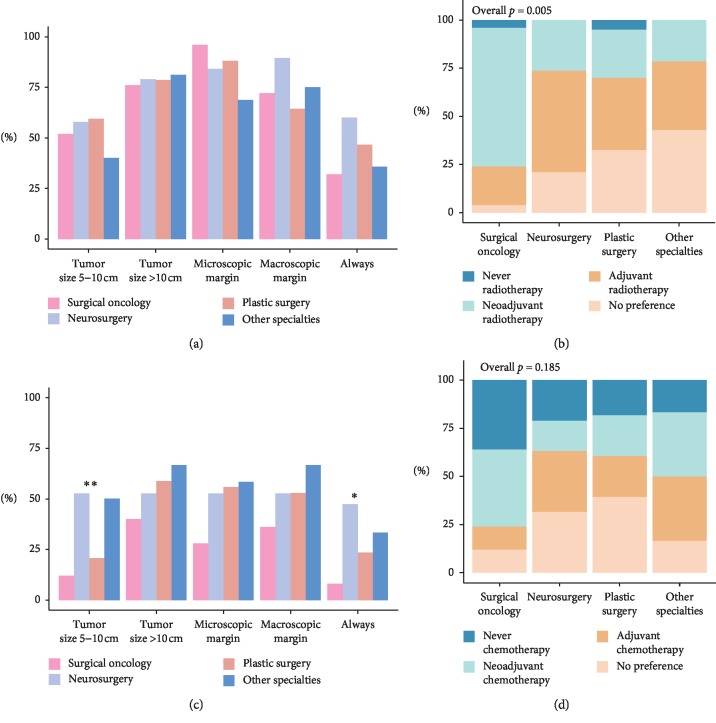
Use of multimodal therapy. (a) Percentage per surgical subspecialty of indications for radiotherapy. (b) A preferred sequence of radiotherapy per surgical subspecialty. (c) Percentage per surgical subspecialty of indications for chemotherapy. (d) A preferred sequence of chemotherapy per surgical subspecialty. *p* values: ^*∗*^ ≤ 0.05, ^*∗∗*^ ≤ 0.01.

Opinions of indications for the use of radiotherapy in localized disease did not differ significantly among surgical subspecialties (all *p* > 0.05, Figure 4). While opinions were divided on whether to use radiotherapy in tumors 5–10 cm of size, 78% of respondents would advise radiotherapy in patients with tumors larger than 10 cm of size. The microscopic positive margin was regarded as an indication for radiotherapy by the majority of respondents (86%), and by an even larger proportion of the surgical oncologists (96%). Forty-three percent of respondents are of the opinion that radiotherapy is routinely indicated in any localized MPNST. A preferred sequence of radiotherapy in any localized MPNST differed significantly among surgical subspecialties (*p* < 0.05). Surgical oncologists preferred neoadjuvant administration (72%), while other subspecialties either preferred adjuvant administration (36–53%) or had no preference (21–43%).

### 3.5. Chemotherapy

Overall, respondents felt that chemotherapy was usually not indicated in localized disease (Figure 4). Only tumor sizes larger than 10 cm (54%) and macroscopically positive margins (51%) were regarded as an indication by more than half of all respondents while tumor sizes 5–10 cm were seen as an indication for the use of chemotherapy by 29% of respondents, neurosurgeons and “other” surgical subspecialties more commonly viewed this as an indication for its use (*p* < 0.05). A total of 26% of all respondents were of the opinion that chemotherapy should always be used in localized disease; this differed significantly among surgical subspecialties (*p* < 0.05). Neurosurgeons most commonly recommended the latter (47.4%). A preferred sequence of chemotherapy in any localized MPNST did not differ between surgical subspecialties (*p* > 0.05), but no consensus was present. Overall, 24% of respondents did not see a role for chemotherapy in any localized MPNST.

## 4. Discussion

In patients who are referred for soft tissue tumors that are possibly MPNSTs, the reported use of preoperative imaging studies and biopsies differs between surgical subspecialties; the vast majority of surgical oncologists routinely perform both. Some surgical considerations such as the extent of resection margins for the preservation of function in selected cases differ within surgical subspecialties. Moreover, assumed indications for the use of radiotherapy and chemotherapy in localized MPNST differ among surgical subspecialties, as well as their ideal timing of administration.

### 4.1. Preoperative Diagnostics in MPNST

Ideally, MPNSTs are resected with a wide margin to obtain an R0 margin [4, 10, 21, 22]. As a result, surgery can be very disabling, underlining the need for correct preoperative diagnosis as benign nerve sheath tumors can be resected without margins. Additionally, obtaining a preoperative diagnosis facilitates the opportunity to administer preoperative radiotherapy or chemotherapy. Therefore, guidelines for treating STS and NF1 both recommend performing MRI imaging and core needle biopsies to obtain a histopathological diagnosis [21–23]. Although radiological features and presenting symptoms are not specific for malignancy, some general indications should make surgeons aware of potential malignancy. Irregular shape and border, lobed aspect, cystic changes, heterogeneous structure, absence of a target sign (distinctive for neurofibromas), and peritumoral edema on MRI may indicate malignant transformation in MPNSTs [8, 9, 24]. Tumors larger than 5 cm or deep to the fascia definitely justify imaging and biopsy [21, 23]. However, preoperative identification of malignancy in NF1 patients is particularly difficult, as atypical and plexiform neurofibromas can present similarly to MPNSTs. Recent research has shown that FDG-PET scans can be helpful in distinguishing malignant from benign lesions, differentiating MPNSTs from neurofibromas with an 80% specificity and almost 100% sensitivity [25, 26], which is why an NF1 consensus does recommend performing it [22]. Others have shown that diffusion-weighted imaging sequences in MRI can differentiate malignancy with 100% specificity; however these techniques are not standard of care in many centers [24]. As neurosurgeons see neurofibromas commonly, it may explain the high proportion of neurosurgical respondents performing FDG-PET scans preoperatively. While surgical oncologists more commonly adhere to guidelines recommending core needle biopsies as preferred biopsy [21–23], a larger proportion in other subspecialties favors open biopsies as well. If an open biopsy were to be considered, ideally, the same surgeon performing the tumor excision should execute the biopsy as the risk of tumor spread is substantially higher [21–23]. Excisional biopsy can also be considered for superficial tumors <3 cm, as this may be most conventional [21, 22]. Differences in preferred biopsy technique between subspecialties may therefore possibly be explained by specialty bias. Fine needle aspirations are discouraged in MPNSTs as they have a high risk for uncertain diagnoses because of small specimen sizes [21–23, 27].

### 4.2. Surgical Treatment in MPNST

Complete surgical excision with wide margins is the routine treatment of choice [4, 10, 21, 22]. Nonetheless, even when obtaining R0 margins, MPNSTs can recur [2–4, 15, 16]. Some authors even propose that fresh frozen coupes are necessary intraoperatively [2, 3, 28]. There is a possibility that as MPNSTs have their perineural origin, skip lesions may be present along the nerve of origin [28]. Respondents to this survey also felt that resecting a longer course of the nerve may, therefore, be beneficial, encouraging future studies to evaluate this in depth. Moreover, while R1 resections are associated with a higher likelihood of recurrence, several large MPNST series have not shown that R1 resections are associated with worse survival compared to R0 resections [4, 6, 10]. This indicates a potential role for operating with closer margins in order to preserve function without altering a patient's prognosis [29]. For instance, tumors in the brachial plexus may be adequately treated with epineural dissection and nerve reconstructions avoiding the need for a forequarter amputation [30]. Contrarily, 42% of respondents to this survey would never perform less extensive resections even if free surgical margins were not presumed feasible. Function preservation was also not considered preoperatively by almost 30% of surgeons. However, considering it in an early stage of treatment may be beneficial, as long-term disabilities may be minimized since localized MPNSTs do have a median survival of 5–8 years [5, 10]. Combining knowledge of reconstructive possibilities by reconstructive and nerve surgeons as an addition to oncological resection margins may improve the delicate balance between oncological and functional outcomes. Such a multidisciplinary approach by these surgical specialties may also optimize the preoperative surgical planning for the extent of the resection to preserve functional anatomy using planned positive margins, or going wider and resecting functional structures beyond the reconstruction tools of the plastic surgeons. Currently, functional reconstructions are uncommonly performed in STS patients, especially those requiring nerve reconstruction, even though outcomes can be very satisfactory [31].

### 4.3. Multimodal Treatment in MPNST

To date, no study has yet demonstrated that MPNSTs should be treated differently than other high-grade STS [13, 18]. As such, MPNST treatment guidelines grossly follow general STS guidelines [21, 23]. However, even in large dedicated sarcoma centers, the use of chemotherapy and radiotherapy differs significantly [18]. Radiotherapy was considered by most respondents in tumors sizes >10 cm and positive surgical margins. This is in concordance with findings in another survey on multimodal treatment in STS and STS guidelines [21, 32]. Although surgical oncologists clearly preferred the neoadjuvant administration of radiotherapy, others did not. Neoadjuvant administration did prove in one trial to require a lower dosage of radiation, which eventually resulted in lower long-term morbidity at the price of increased postoperative complications [33, 34]. However, neoadjuvant radiotherapy may complicate possible nerve reconstruction, and fibrous tissue will always have to be removed to create a vascularized wound bed for nerve regeneration [35]. As such, the differences in opinion on preferred timing may also be related to specialty bias. Indications for the use of chemotherapy in localized MPNSTs and STS, in general, are conflicting, as reflected by responses to this survey. Thus far, trials and meta-analyses have not been able to provide definitive conclusions on the beneficial effect of perioperative chemotherapy in STS as observed effects are relatively small [36–39]. Preliminary results from a recent randomized trial did, however, show a positive effect for localized high risk (high-grade, large, and deep-seated) extremity STS on both overall survival and disease-free survival [13]. For MPNSTs, chemotherapy regimens should ideally involve an anthracycline-based regimen, such as doxorubicin, in combination with ifosfamide [13, 14, 40, 41]. Preferred timing of chemotherapy administration has not been studied thoroughly, but several hypotheses exist favoring neoadjuvant therapy translated from research in breast cancer. This includes earlier initiation of systemic therapy, possible downstaging of the tumor, and eliminating micrometastases before exposure to wound-healing cytokines triggered by operation [41–43]. However, these theories have not yet been proven in STS. Unfortunately, studies show that MPNSTs are relatively chemoresistant, possibly more so in NF1 patients [41, 43]. Some smaller studies suggest MPNST can respond well to chemotherapy, but exact populations that may respond are to be elucidated [44, 45]. More clinical studies are warranted to find tumor-tailored noncytotoxic treatments, alas, so far, none have been proven effective in MPNSTs [46]. As the debate on the exact role of multimodal therapy in localized disease is still evolving, advantages and disadvantages are to be discussed with patients after general discussion in a multidisciplinary tumor board. Several STS calculators have been proved useful for decision-making [47, 48]. Again, by including both oncological and reconstructive surgeons when planning patient treatment for localized disease, an ideal strategy can be obtained for the timing of multimodal therapy as opposed to oncological resection and possible functional reconstruction.

### 4.4. Strengths and Limitations

Limitations to this study are partially inherent to the survey methodology. Respondent bias should always be taken into account as only interested surgeons will fill out the survey. Furthermore, selection bias may be present as we restricted our survey distribution to a certain list of surgical societies, thereby excluding physicians that are not members of these societies. This study is, however, strengthened by the combination of respondents with experience in both sarcoma and peripheral nerve surgery. As patients will present themselves to several surgical subspecialties, it is important that knowledge and experience are exchanged, more so when practice variation is present. Partially, as several elements of MPNST treatment have not been proven by high-level evidence, of which some will likely never be because of their low incidence. Future studies should be encouraged in combining data from several subspecialties and to further explore the ideal combination of surgical treatment and function preservation and the role of multimodal treatment. Multidisciplinary approaches are essential for optimal treatment of MPNSTs, possibly including collaboration of surgical oncologists, nerve surgeons, and reconstructive surgeons. In turn, consensus guidelines among all specialties treating MPNSTs can and should be made.

## 5. Conclusion

While a consensus among surgical oncologists is more apparent in preoperative diagnostics, this differs between surgical subspecialties. Some disagreement exists as well within subspecialties on less extensive resections in selected cases for function preservation. While surgeons agree on some indications for radiotherapy, the preferred sequence of radiotherapy differed between surgical subspecialties and within subspecialties other than oncologic surgery. Chemotherapy seems less popular in localized disease, and indications for its use lack consensus among surgeons. Differences between surgical subspecialties are likely caused by specialty bias, and combining knowledge between surgical subspecialties may further ameliorate patient outcomes.

## Figures and Tables

**Table 1:** Demographical data of survey respondents.

Variable (N)	Oncologic surgery	Neurosurgery	Plastic surgery	Other specialties	*p*
	30	30	85	29	
Experience					
Mean (SD)	15.64 (9.31)	13.26 (8.64)	13.49 (9.81)	15.64 (10.13)	0.603
<10 years	28.6%	37.0%	43.1%	36.0%	0.585
10–20 years	50.0%	37.0%	34.7%	28.0%	
>20 years	21.4%	25.9%	22.2%	36.0%	

Fellowship training					
Sarcoma	81.5%	0.0%	2.8%	8.0%	**<0.001**
PNS	0.0%	55.6%	29.2%	56.0%	
Sarcoma & PNS	3.7%	0.0%	2.8%	0.0%	
Other or none	14.8%	44.4%	65.3%	36.0%	

Annual caseload					
0-1	18.5%	50.0%	70.4%	66.7%	**<0.001**
2-3	22.2%	34.6%	22.5%	12.5%	
3–5	33.3%	15.4%	2.8%	12.5%	
>5	25.9%	0.0%	4.2%	8.3%	

Tumor sites operated					
Intracranial	0.0%	34.6%	0.0%	0.0%	**<0.001**
Head & neck	18.5%	42.3%	14.1%	8.3%	**0.007**
(Para)spinal	22.2%	76.9%	1.4%	4.2%	**<0.001**
Superficial thoracic	55.6%	34.6%	8.5%	8.3%	**<0.001**
Intrathoracic	37.0%	15.4%	0.0%	0.0%	**<0.001**
Abdominal	74.1%	23.1%	5.6%	4.2%	**<0.001**
Retroperitoneal	74.1%	46.2%	4.2%	0.0%	**<0.001**
Pelvic	81.5%	38.5%	1.4%	8.3%	**<0.001**
Extremities	85.2%	84.6%	93.0%	75.0%	0.136
Brachial plexus	37.0%	65.4%	35.2%	41.7%	0.059

N: number, PNS: peripheral nerve surgery, SD: standard deviation.

## Data Availability

The data used to support the findings of this study are available from the corresponding author upon request.

## Additional Points

*Precis*. Variation in treatment exists among surgeons operating MPNSTs, but more so between subspecialties. Combining expertise from several surgical subspecialties may be beneficial for outcomes and filling knowledge gaps in MPNSTs.

## Supplementary Materials

Supplementary Materials Survey MPNST surgery.
