# Supplementary material for: Oncological Treatment Considerations Differ across Surgical Subspecialties Treating Malignant Peripheral Nerve Sheath Tumors: An International Survey
Source: Sarcoma. 2020 Feb 27;2020:6406439. doi: 10.1155/2020/6406439 (PMC7064831; doi:10.1155/2020/6406439)
Supplement: Supplementary Materials — Survey MPNST surgery. [file 6406439.f1.docx]

**Survey MPNST surgery**

- Only one box can be selected
- Multiple boxes can be selected

… Free text

<https://www.nvpc.nl/jh/surveys/?s=PMTEJRY4LA>

**General introduction**

Thank you for participating in this questionnaire about the treatment of malignant peripheral nerve sheath tumors. Please keep in mind that the questions regard your personal opinion on optimal treatment of localized disease.

**1) Select your surgical subspecialty**

- Oncologic Surgery
- Neurosurgery
- Plastic Surgery
- Orthopedics (other than oncologic surgery)
- General Surgery (other than oncologic surgery)
- ENT
- Maxillofacial
- Thoracic Surgery

**2) Select your country of practice**

- Dropdown list of all countries

**3) How many years ago did you finish your surgical training? Please provide your answer in full years**

...

**4) Are you subspecialized in peripheral nerve surgery?**

- Yes
- No

**5) Are you a fellowship trained: Multiple answers can be selected**

- Peripheral nerve surgeon
- Sarcoma surgeon
- Other or none

**6) On average, how many MPNST cases do you operate annually?**

- 0-1
- 2-3
- 3-5
- >5

**7) Select the tumor locations you operate. Multiple answers can be selected.**

- Intracranial
- Extracranial head & neck
- (Para-) Spinal
- Superficial Thoracic
- Intrathoracic
- Abdominal
- Retroperitoneal
- Pelvic
- Extremities (excluding plexus)
- Brachial Plexus

**8) Do you attempt to distinguish MPNSTs from its benign counterparts AND other sarcomas preoperatively?**

- Generally not, but we do use radiology and/or biopsy
- With the use of radiology
- With the use of biopsy
- Using both biopsy and radiology

**9) What type of imaging do you generally use preoperatively? Multiple answers can be selected.**

- MRI
- CT-thorax
- FDG-PET
- Other

**10) If there is a suspicion for an MPNST, what type of biopsy do you prefer using?**

- Open biopsy
- Core needle biopsy
- Fine needle aspiration
- Stereotactic biopsy
- Ultrasound-guided biopsy
- Other
- Generally no biopsy is performed: …
- Do not know

**11) When deciding the use of radiotherapy, which of the following patient or tumor characteristics would prompt you to use radiation?**

a) Primary tumor size 5-10 cm

b) Primary tumor size >10 cm

c) Age <50 years

d) Microscopic margin

e) Macroscopic margin

f) In principle, we always use radiotherapy

**12) What is your preferred sequence of radiotherapy when used?**

- Neoadjuvant
- Adjuvant
- No preference
- We never use radiation in localized disease

**13) When deciding the use of systemic chemotherapy in localized disease, which of the following patient or tumor characteristics would prompt you to use systemic chemotherapy?**

a) Primary tumor size 5-10 cm

b) Primary tumor size >10 cm

c) Age <50 years

d) Microscopic margin

e) Macroscopic margin

f) In principle, we always use chemotherapy

**14) What is your preferred sequence of chemotherapy in localized MPNSTs when used?**

- Neoadjuvant
- Adjuvant
- No preference
- We never use systemic chemotherapy in localized disease

**15) What is the most common non-oncologic postoperative complication after MPNST surgery?**

- Neuropathic pain, dysesthesia, allodynia, or cold intolerance
- Motor disability
- Sensory deficiency
- A combination of neuropathic pain and neurologic deficit
- None of the above

**16) In your clinic, how often does a patient present with a functional motor deficit postoperatively?**

… %

**17) In your clinic, how often does a patient present with neuropathic pain postoperatively?**

… %

**18) Do you always consider preservation of function preoperatively? If ‘sometimes’, please explain briefly.**

- Yes
- No, oncologic resection is always more important
- Sometimes: …

**19) Given that oncological resection of some MPNSTs can cause large functional deficits, are there cases that you resect less of the tumor in order to preserve functionality?**

- Yes, sometimes
- Yes, but only when free margins are not presumed possible
- No, never

**20) Do you operate MPNSTs together with a peripheral nerve surgeon?**

- Yes
- No
- Sometimes

**21) Do you use intraoperative nerve conduction testing when operating MPNSTs?**

- Yes
- No
- Sometimes

**22) Intraoperatively, do you always search for the nerve from which the MPNST originated? If ‘no’, please explain briefly.**

- Yes
- No: …
- Sometimes

**23) What is your preferred treatment of the transected nerve? If ‘other’, please explain briefly.**

- Nothing
- Bury in bone/muscle/vein
- Closure end with adhesive or epineural graft
- Neurorrhaphy
- Targeted Muscle Reinnervation
- Other: …

**24) Do you perform functional reconstruction (i.e. muscle/nerve/tendon reconstructions) if a motor deficit is anticipated?**

- Never
- Generally not
- Sometimes
- Always

**25) Do you consider functional reconstruction (i.e. nerve reconstruction or innervated skin flap) if a sensory deficit is anticipated?**

- Never
- Generally not
- Sometimes
- Always

**26) What is your preferred timing of functional reconstruction after initial surgery?**

- Direct regardless of radiotherapy
- Direct if no postoperative radiotherapy will administered, otherwise after radiotherapy
- Delay of 3 months
- Delay of 6-12 months
- I do not consider MPNST patients eligible for functional reconstruction

**27) What functional reconstructions do you consider as a possibility? Multiple answers can be selected.**

- None
- Nerve reconstruction
- Nerve transfer
- Tendon transfer
- Free functional muscle transfer
- Do not know

**28) In case ANY form of functional deficit is present (i.e. loss of sensibility or any motor function loss), select factors that would prevent you from considering functional reconstruction in a patient. Multiple answers can be selected.**

- The general low survival of MPNSTs
- A non-extremity MPNST
- Use of radiotherapy
- Slow nerve regeneration
- Slow rehabilitation of function
- The nerve(s) from which an MPNST originated are ‘sick’ and cannot be used
- Other (provide answers in text field below)
- None

**29) What should be the median survival of a patient, in your opinion, before considering functional reconstruction? Please provide your answer in full years.**

…

**30) The following situation is present: An MPNST has been resected with clear margins. MPNSTs grow ‘perineurally’, commonly recur, and metastasize frequently, do you believe that this may be due to microscopic satellite lesions along the nerve and by resecting the originating nerve as proximal and/or distally as possible could have beneficial effect?**

- Yes
- No

**If you have any questions regarding this survey or interest in collaboration for further research in MPNSTs, please leave your email address in the following text field.**

…
